# Vascular cambium regeneration and vessel formation in wounded inflorescence stems of Arabidopsis

**DOI:** 10.1038/srep33754

**Published:** 2016-09-21

**Authors:** Ewa Mazur, Eva Benková, Jiří Friml

**Affiliations:** 1 Department of Cell Biology, Faculty of Biology and Environmental Protection, University of Silesia in Katowice, 40-032 Katowice, Jagiellońska 28, Poland; 2 Mendel Centre for Plant Genomics and Proteomics, Central European Institute of Technology (CEITEC), Masaryk University (MU), CZ-62-500 Brno, Czech Republic; 3 Institute of Science and Technology (IST), 3400 Klosterneuburg, Austria

## Abstract

Synchronized tissue polarization during regeneration or de novo vascular tissue formation is a plant-specific example of intercellular communication and coordinated development. According to the canalization hypothesis, the plant hormone auxin serves as polarizing signal that mediates directional channel formation underlying the spatio-temporal vasculature patterning. A necessary part of canalization is a positive feedback between auxin signaling and polarity of the intercellular auxin flow. The cellular and molecular mechanisms of this process are still poorly understood, not the least, because of a lack of a suitable model system. We show that the main genetic model plant, Arabidopsis (*Arabidopsis thaliana*) can be used to study the canalization during vascular cambium regeneration and new vasculature formation. We monitored localized auxin responses, directional auxin-transport channels formation, and establishment of new vascular cambium polarity during regenerative processes after stem wounding. The increased auxin response above and around the wound preceded the formation of PIN1 auxin transporter-marked channels from the primarily homogenous tissue and the transient, gradual changes in PIN1 localization preceded the polarity of newly formed vascular tissue. Thus, Arabidopsis is a useful model for studies of coordinated tissue polarization and vasculature formation after wounding allowing for genetic and mechanistic dissection of the canalization hypothesis.

Development and patterning of vascular tissue require signaling and directional flow of the plant hormone auxin^1^,^2^,^3^. Two models to explain the pattern of vessel formation have been proposed. One model is based on the reaction-diffusion hypothesis^4^ and the other, the so-called canalization hypothesis, proposes a directional auxin flow as the main signal for vascular tissue development^5^,^6^.

The auxin flow direction has been shown to depend on the asymmetric position of the PIN-FORMED (PIN) auxin transporters at the plasma membranes of transporting cells^7^,^8^,^9^,^10^,^11^,^12^,^13^,^14^. Development of many plant organs, such as lateral roots or cotyledons, is strictly correlated with the establishment of local PIN-dependent auxin gradients that precede cell divisions and differentiation processes^15^. The constitutive recycling of PIN proteins from and to the plasma membranes that involves clathrin-dependent endocytosis^16^ allows dynamic changes in PIN localization and increased stabilization of PIN proteins at the plasma membrane in response to auxin^17^,^18^. Changes in PIN localization and tissue polarity in response to auxin that are presumably related to the directional vascular tissue patterning have been observed and modelled^1^,^2^,^3^,^19^,^20^. For example, when the auxin flow direction is interrupted (i.e. by wounding), the subcellular position of PIN proteins changes and the cell polarity is established de novo gradually concentrating the auxin flow for the formation of new auxin channels^2^,^3^. The auxin-dependent canalization is supported experimentally by studies on leaf vein patterning and on the role of the genes encoding the auxin response factor *MONOPTEROS (MP*) and the auxin transport protein *PIN1*^21^. Both genes undergo the dynamic expression and subcellular positioning of PIN1 transporters that gradually change from nonpolar to polar, indicating the auxin flow direction during the vascular patterning^21^.

Vascular tissue disruption in experimental systems leads to regeneration processes. In nonwoody dicotyledonous plants, vasculature is regenerated in the wound neighborhood of primary tissues^2^,^3^,^5^,^6^,^22^. New vessels are arranged around the wound according to the presumable new auxin flow direction or form either the so-called bypass strands directly through the wound^22^ or bridges between the neighboring vascular bundles^23^. For decades, analysis of vascular pattern reconstruction from incised vascular cambium during its regeneration was restricted to trees and plants with well-developed secondary tissue architecture and thick cambium^24^,^25^,^26^. Thus far, cambium and its activity were analyzed mainly in trees^27^,^28^,^29^,^30^ and the results revealed an important role for cambium in secondary xylem formation and thickening of woody plants in the nondisturbed development. Because of the difficulties in using woody plants as a model system^31^, mechanisms of cambium regeneration are still poorly understood. However, in trees, vascular tissue regenerates very fast in the wounded areas and this process is accompanied by the development of enlarge amounts of callus, numerous shortening anticlinal divisions of cambial cells and intrusive growth^32^,^33^. Thus, following the canalization concept, regenerated vessels organized with threads of short cells above or around the wound^34^,^35^, support the emergence of auxin channels according to the new auxin transport direction in incised regions. In some instances, e.g. after wounding, the so-called circular vessels develop^36^,^37^,^38^. Circular vessels occur in the form of rings and are presumably induced as a consequence of the circular auxin flow route and the establishment of the circular polarity of individual cells that dedifferentiated into this type of vessels^36^. Accordingly, circular vessels develop as a response of individual cells to the auxin flux rather than to the local auxin concentration.

Thus far, studies on vascular cambium regeneration and accompanying changes in auxin distribution, flow directionality, and cellular polarity of PIN transporters have been hampered by the inability to induce and follow these processes in Arabidopsis (*Arabidopsis thaliana*), making it impossible to use large collections of genetic material available for this model. Nonetheless, artificial weights applied to the apical parts of immature inflorescence stems of Arabidopsis (stems with primary tissue architecture) increase the basipetal auxin transport, stimulate stem growth, and promote secondary growth in basal parts of these stems^39^. Secondary tissue architecture in mechanically stimulated immature inflorescence stems of Arabidopsis develops in a very short time, namely 3 days^39^ or 6 days^40^, which is much faster than in hypocotyls^41^,^42^,^43^ or mature inflorescence stems^44^,^45^,^46^,^47^,^48^. Therefore, decapitation of floral parts and weight application not only allow performing the experiments on much younger plants, but also to analyzing processes of vasculature regeneration with the whole complexity of the “tree-like” tissues^40^,^49^, hence, giving the opportunity to answer still open questions on the auxin-mediated canalization hypothesis in models with secondary growth and functional cambium.

Here, we induced wounding in Arabidopsis inflorescence stems with active cylinder of vascular cambium, and analyzed the correlations between auxin distribution, tissue polarization, and formation of PIN1-mediated auxin channels during vascular tissue regeneration.

## Results

### Histological analysis of vascular tissue regeneration after wounding

**Figure 1:**
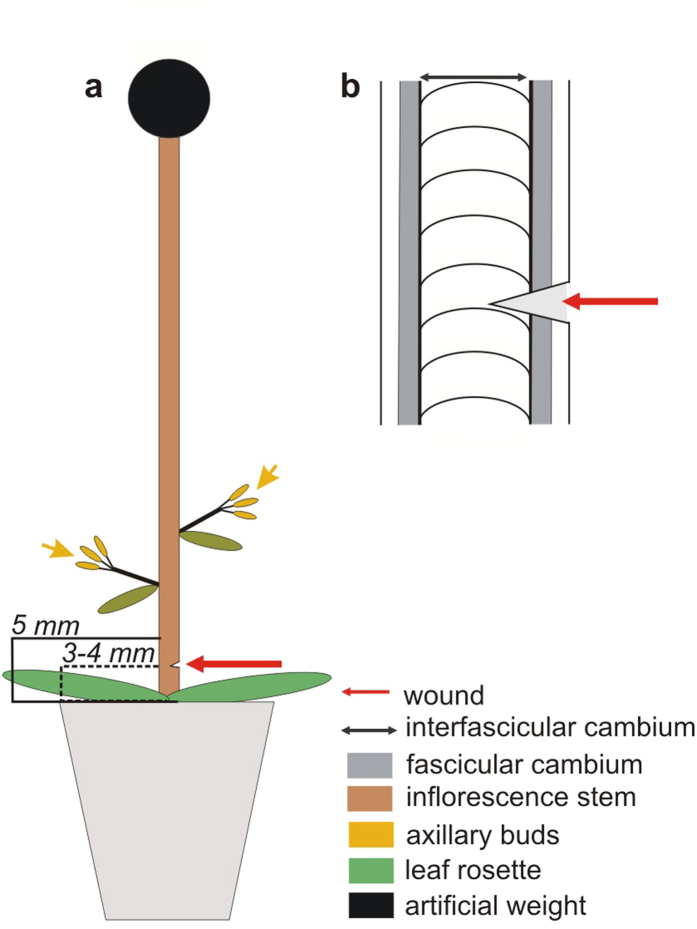
Design of the experiments with the wounded stems. **(a)** Inflorescence stems with secondary tissue architecture in the basal parts were wounded (transversal cut) 3 to 4 mm above the rosette. The stem apex remained covered with the artificial weight (2.5 g) during the experiment. The axillary buds above the rosette were not removed and were an endogenous auxin source. **(b)** Transversal incision was made with a razor blade precisesly in the sectors with secondary tissue architecture, to interrupt the longitudinal vascular cambium continuum (primary fascicular cambium belonging to vascular bundles + interfascicular cambium developed from interfascicular parenchyma cells) and secondary tissues.

Plants were grown under special conditions, according to the method previously described^39^ and modified^40^ (Fig. 1a). An artificial weight (2.5 g) was applied to immature stems with decapitated apical parts (Fig. 1a). Axillary buds above the leaf rosette were not removed (Fig. 1a) and were a source of endogenous auxin (see also Supplementary Figs S1 and S4). The basal parts of the weight-applied stems were cut transversally to interrupt the longitudinal continuum of vascular cambium and secondary tissues, necessary to analyze the vasculature regeneration in wounded areas (Fig. 1b).

**Figure 2:**
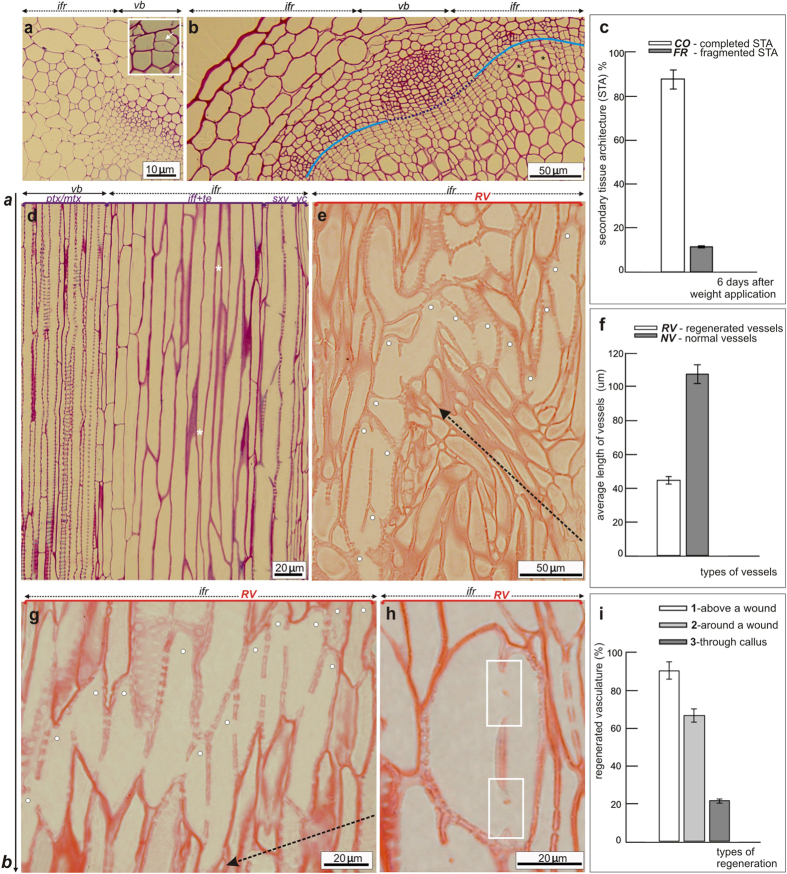
Histological analysis of differentiated vessels. **(a)** Unwounded immature stem, 1 day after artificial weight application, was characterized by primary tissue architecture, namely vascular bundles (*vb*) were separated by interfascicular regions *(ifr*) - sectors of interfascicular parechyma cells. The artificially applied weight stimulated periclinal divisions of interfascicular parenchyma cells (*inset, arrow*) and their dedifferentiation into cambial cells. **(b)** Transition from primary to secondary tissue architecture (STA) occurred 6 days after the weight application. Close ring of vascular cambium developed from fascicular cambium of vascular bundles (*vb*) and interfascicular cambium in interfascicular regions (*ifr*). Both of the cambia are indicated by lines. Numerous periclinal divisions stimulated development of secondary phloem and xylem, outside and inside cambium, respectively. Asterisks indicate differentiated vessels. **(c)** Statistical analysis showing very high efficiency of the transition to STA in immature stems after weight application. **(d)** Tangential view – 6 days after weight application. Secondary xylem vessels (*sxv*) developed along the apical-basal stem axis, indicated by (**a,b**) arrow, as conductive longitudinal vessel strands. Vessel elements were connected by open perforations localized on their opposite ends and characterized by an enlarge lumen and thick secondary cell walls [in comparison to primary xylem vessels, protoxylem (*ptx*) /metaxylem (*mtx*), found in vascular bundles (*vb*)]. Mature vessels in controls adjacent to interfascicular fibers (*iff*) and other tracheary elements (*te*) differentiated from vascular cambium (*vc*) in the interfascicular regions (*ifr*). **(e)** In incised stems, 6 days after wounding, regenerated vessels (*RV*) were arranged in threads of short cells marked by dots and often developed around the wound circumventing the incision edge. **(f)** Regenerated vessels were over two-fold shorter than normal vessels in controls. (**g**) Possible vessel strands often developed above the wound, more or less parallel to the incision edge. Vessel elements, marked by dots, were connected by perforations localized on their lateral cell walls. **(h)** In wounded regions circular vessels differentiated as well and were arranged from two, rarely three, cells connected in closed rings by perforation-like plates or enlarged bordered pits on their lateral cell walls (*boxed sectors*). **(i)** Vasculature regenerating mainly above and around a wound (91% and 67% of all analyzed stems, respectively) and also directly through the callus, formed “bypass” vessel strands. Statistical evaluations were done with unpaired Student *t*-test, *P* < 0.05. Broken arrows indicate wound.

The secondary growth in weight applied stems of Arabidopsis has been described previously in detail^40^, therefore we present here the transition from primary to secondary tissue architecture (Fig. 2a,b) only as an introduction to events observed during vasculature regeneration. The applied weight triggered dedifferentiation of interfascicular parenchyma cells in sectors localized between the vascular bundles and their periclinal divisions (Fig. 2a). Finally, the vascular cambium developed into a closed ring on the stem circumference and produced secondary vascular tissues (Fig. 2b). Thus, 6 days after the weight application, secondary tissue architecture was observed in the basal parts of the previously immature Arabidopsis stems. Almost 90% of all analyzed stems were characterized by such a tissue arrangement (Fig. 2c).

As controls for the weight-applied stems, mature inflorescence stems (>25 cm tall) were also analyzed (Supplementary Fig. S2). Such plants were obtained in more than 2 months, showed secondary growth in narrow sectors of the basal parts (2 to maximum 3 mm thick), but fully closed ring of vascular cambium occurred in approximately 50% of the mature stems. Thus, in many of the old stems (more than 40%), secondary growth was fragmented, i.e. no secondary tissues developed on the whole stem circumference. Various cambial phenotypes, such as rays and intrusively grown fusiform cambial cell, were not found. Hence, for all experiments, we used young stems with applied weights.

Histological analysis of both nonincised controls and wounded stems revealed significant differences in vessels organization (Fig. 2d,g). A series of semi-thin sections were examined to get an idea about the vessel strand arrangements, but only one representative section was selected as illustration. In unwounded controls, vessels were organized in vertical strands parallel to the longitudinal stem axis and reflected the arrangement of cambial cells (Fig. 2d). Open perforations were localized at the opposite (apical-to-basal) ends of the neighboring cells that connected with each other in conductive strands (Fig. 2d). The developed vessels were always adjacent to other tracheary elements and emerged as cells with relatively enlarge lumen and thick secondary cell walls (Fig. 2d).

In wounded stems of Arabidopsis, the regenerated vessels were much shorter (Fig. 2e–g) than those developed in unwounded controls. Statistical analysis revealed that regenerated vessels were almost two-fold shorter than normal vessels in controls (45 μm versus 109 μm average cell length) (Fig. 2f). As a consequence, the regenerated vessels were arranged in threads of short elements that either circumvented the wound (Fig. 2e) or developed above the wound (Fig. 2g). In the latter case, the vessel arrangement was more or less parallel to the cut, but not parallel to the main stem axis. In the regenerated vessels, the perforations were localized mainly on the lateral cell walls and connected individual cells in vessel strands (Fig. 2e,g).

An interesting regeneration manner was observed in deeply wounded stems, few days after wound (DAW), in which the so-called circular vessels developed (Fig. 2h). In the case of the circular vessels, lateral openings were observed that closed into a ring (Fig. 2h).

The histological analysis revealed different ways of the vascular cambium regeneration and the gradual vasculature reconstruction in wounded Arabidopsis stems (Fig. 2i).

### Vascular tissue regeneration monitored by *AtHB8* expression

**Figure 3:**
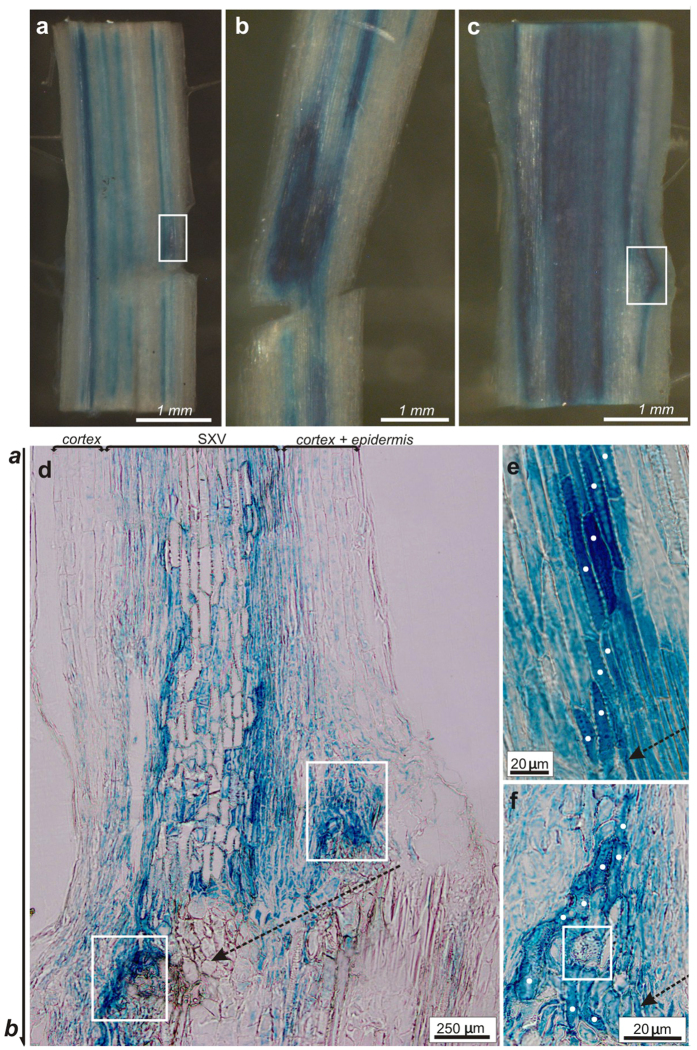
Expression of the *AtHB8* vascular marker in wounded stems. **(a–c)** The *AtHB8* expression in wounded stem tissues. (**a**) Starting from day 1 after wounding (DAW), the gene activity was found in the outer tissues, closely to the wound (*boxed sector*) and increased over the next days. Positive GUS reaction in these tissues is indicated by arrow. **(b)** Very strong gene expression was extended in tissues along the whole upper edge of the wound and around, starting from 4 DAW. **(c)** High *AtHB8* expression was also found in regenerated vessel strands, forming “bypasses” directly through the callus in 13 DAW (*boxed sector*). The “bypasses” connected interrupted tissues in wounded areas. **(d)** Semi-thin section through an incised stem, 4 DAW. *AtHB8* expression in differentiating secondary xylem vessels (*SXV*) emerging distantly from the wound, parallel to the longitudinal stem axis indicated by **a,b** arrow, and in regenerating vasculature in the neighborhood of a wound, both above and around a wound (*boxed sectors*). Gene activity was always very high in cells differentiating into vessels. **(e,f)** Semi-thin sections from wounded areas show development of new vasculature, 4 DAW. Regenerating vessels, marked by dots, were found in the neighborhood of the upper edge of the wound (**e**) or around a wound (**f**). *AtHB8* expression was very strong in all cells differentiating into new vessels as compared to the surrounding neighbours. In places close to the wound, circular vessels differentiated as well (*boxed sector*). Broken arrows indicate wound. **(d–f)** show one representative semi-thin section selected from multiple series of analyzed sections, forming the basis for the delineation of putative vessel strands.

As the *AtHB8* gene is known as a vessel formation marker^50^,^51^, its expression was analyzed to follow cambium regeneration and formation of new vasculature. The *AtHB8::GUS* transgenic line in Wassilevskija (Ws) background^50^ was used. High *AtHB8* gene expression was found in the wounded stem regions (Fig. 3a–c). Primarily, the *AtHB8::GUS* signal was observed in small areas above a wound, 1 day after incision (Fig. 3a) and gradually extended in next 2 days. On day 4 after the transversal cut, the expression of the marker gene was very high in enlarged regions above and around the wound (Fig. 3b). Sometimes, new vasculature developed forming characteristic “bypass” vessel strands, directly through the wounding (Fig. 3c). These “bypass” vessels differentiated from callus cells inside the wound between 10 to 13 days after the incision. Analysis of semi-thin sections confirmed, as expected, the *AtHB8* expression in the regenerating vasculature (Fig. 3d–f). The β-glucuronidase (GUS) signal was higher in differentiating vessels than in the surrounding cells (Fig. 3d–f). The regeneration process was clearly visible starting from day 4 after the cut. New vessels developed both above (Fig. 3e) and around the wound (Fig. 3f). Regenerated vessel elements were sometimes very short (Fig. 3f). Ring-arranged vessels, called circular vessels, developed also adjacent to the incision edge (Fig. 3f). Fully reconstructed vascular tissue was found at later regeneration stages, beginning from day 6 after wounding.

### DR5-monitored local auxin response during vascular tissue regeneration

**Figure 4:**
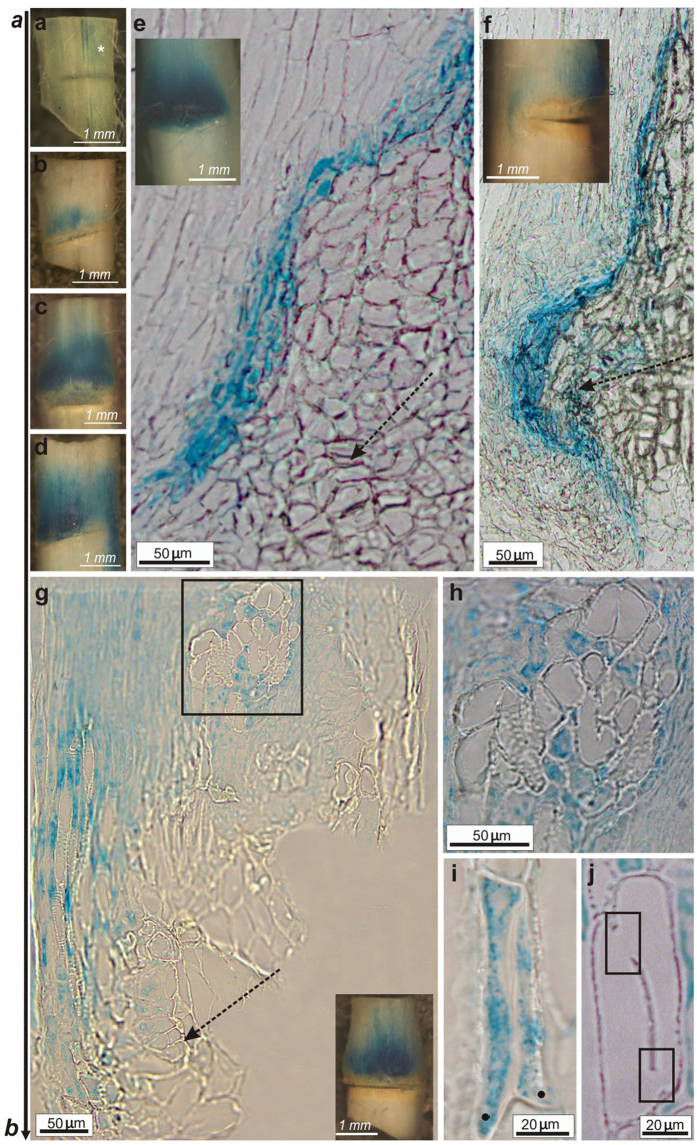
Changes in *DR5::GUS*-monitored auxin responses in wounded stems. **(a)** First 24 h after wounding. Auxin response occurred above and below the wound, the DR5 activity increased slightly in the tissues above the wound, indicated by asterisk. **(b,c)** In days 2 and 3 after wounding, auxin response was high above the wound, in tissues along the upper incision edge and gradually increased in the following days. **(d)** Starting from day 3 after wounding, the auxin response was also very high in the tissues around the wound, circumventing the transversal cut. **(e)** Regenerated vessels developing in the wounding regions with elevated auxin responses (*inset*). Semi-thin sections revealing the emergence of new auxin response-positive channels, often along the upper wound edge from cells with higher DR5::GUS activity than that of the surrounding cells. **(f)** Regenerated vessel strands emerging also in regions with elevated auxin responses around the transversal cut (*inset*). New auxin channels developed here in the wound neighborhood, circumventing the lateral incision edge. Inside the channels, the auxin response was very high. **(g)** Elevated auxin responses in deeply wounded stems (*inset*). Small groups of atypical vessels developed in such places above the wound (*boxed sector*). **(h)** Magnification of the boxed sector. Mature vessels were never arranged in strands, but were characterized by open perforation plates and secondary cell walls and were surrounded by cells with elevated auxin responses. Circular vessels developed here very often. **(i)** Circular vessels developing from two neighboring cells with high DR5 activity inside them. Intrusively growing ends, indicated by dots, were commonly observed in such cells dedifferentiating into circular vessels. **(j)** Mature circular vessels arranged in a closed ring. Individual vessel cells were connected by open perforations/enlarged pits localized mainly on their lateral cell walls (*boxed sectors*). Circular vessels emerged faster among still DR5-positive neighboring cells. Broken arrows indicate wound. **(e–g)** present one representative semi-thin section selected from multiple series of analyzed sections, forming the basis for the delineation of putative vessel strands.

Overall changes in auxin responses after wounding were analyzed in the *DR5::GUS* transgenic line^52^ and the *GUS* expression was followed day by day from day 1 to 12. First, we attempted to analyze auxin responses in wounded areas on transversal sections (Supplementary Fig. S3), that were made around the wound/regenerated vasculature areas. Three and 4 days after incision, auxin responses were observed in almost all wounded tissues (even in cortex and epidermis), but they were poorly recognizable in the nearest neighborhood of the wounds and, thus, vasculature regeneration could hardly be identified in this sectioning plane. In contrast, the longitudinal sections were very informative and revealed that the DR5-monitored auxin responses were not uniform in the wounded areas. The most important changes were observed in the first 4 days (Fig. 4a–d). During the first 24 h after wounding (transversal cut), *DR5::GUS* was expressed both above and below the cut (Fig. 4a). However, during the next 48 h, the auxin response became restricted, with the highest signal in the regions that extended along the upper edge of the transversal cut and increased afterward (Fig. 4b,c). Thus, starting from day 3 after wounding, the highest auxin response was detected not only above, but importantly, also around the cut (Fig. 4d). Moreover, during this period, DR5 activity and elevated auxin responses did not occur in tissues below the cut (Fig. 4d).

Next, we looked more closely at the DR5 signal in cells during vascular tissue formation. Analysis of semi-thin sections revealed vascular tissue regeneration at different positions around the wound. Vasculature often regenerated above the upper wound edge, in the middle parts of the transversal cut (Fig. 4e, inset). In these areas, the auxin response was the highest and the new auxin channels developed not along the longitudinal stem axis, but approximately parallel to the cut (Fig. 4e).

A presumptive regeneration path indicated by the *GUS* expression was also observed around the wound, in areas generally marked by the elevated auxin response (Fig. 4f, inset). The top and bottom sectors of emerged channels were parallel to the longitudinal stem axis, but their middle parts developed around the wound, circumventing the transversal cut edge (Fig. 4f).

**Figure 5:**
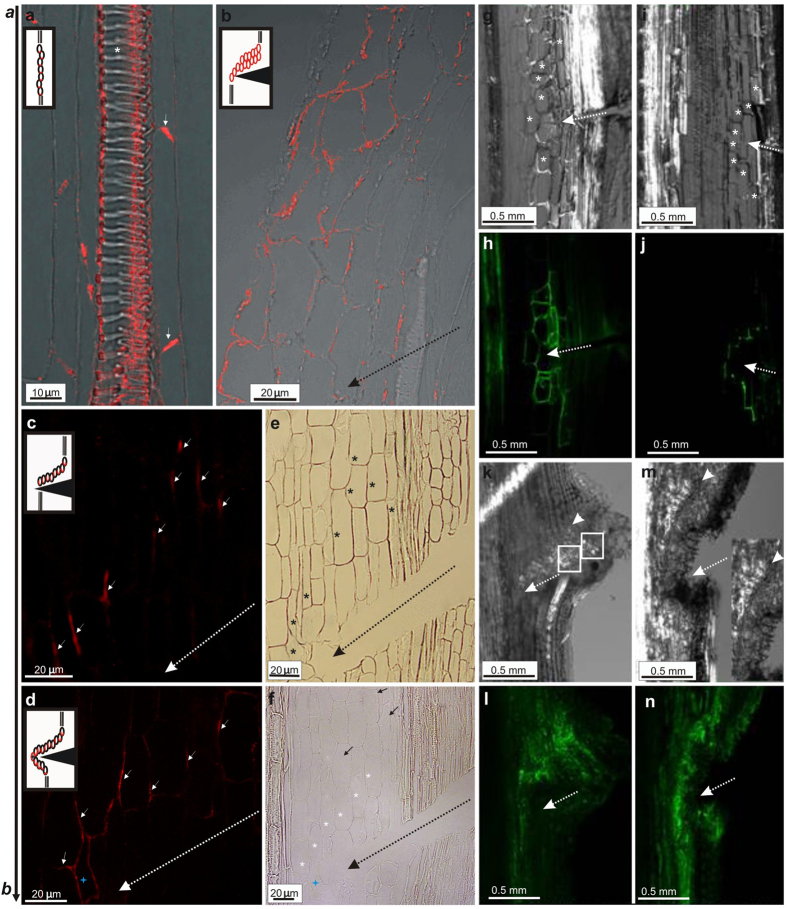
PIN1 polarity establishment in auxin channels. **(a)** Col-0, unwounded control stem. Arrows indicate polar localization of PIN1 auxin transporters on the basal plasma membranes of the cambial cells. Vessels (visible primary xylem vessels indicated by asterisk) developed along the apical-basal stem axis, indicated by **a,b** arrow. **(b)** Day 1 after wounding (DAW). PIN1 position changed in the cells above the wound: PIN1 moved from the basal to the lateral and apical plasma membranes. **(c,d)** Wounded stems, 3 DAW. Arrows and models indicate new established polarity in regions above (**c**) and around the wound (**d**). PIN1 shifted to the lateral plasma membranes of cells in newly emerged PIN1-marked auxin channels. Asterisk indicates apolar localization of PIN1 in the cell in the closest vicinity of the wound. **(e,f)** Bright-field images for tissue arrangement in (**c**,**d**), respectively. Asterisks indicate the cells with changed PIN1 position. Directional divisions of cells in wounded regions visible in (**f**) are indicated by arrows. **(g–j)** *pPIN1::PIN1:GFP* transgenic line, 1 and 2 DAW. (**g,i**) Transmitted light images. Asterisks indicate cells around the wound, where PIN1-GFP was shifted. (**h,j**) GFP visualized PIN1 polarity in the wounded areas. **(h)** First changes in PIN1 polarization were very fast in the wounded areas. PIN1 position shifted from basal to apical and lateral plasma membranes in cells around the wound already in day 1. **(j)** PIN1 polarity was established in 2 DAW. Transporters shifted to the lateral plasma membranes in cells around the wound, starting to form an auxin channel. **(k–n)** *DR5::GFP* transgenic line 3 and 4 DAW. **(k,m)** Transmitted light images. Arrowheads indicate vessels regenerated above the wound and circumvented the wound edge. Inset in (**m**) magnification of the marked vessel strand. Vessels were also found in the outer tissues, next to the callus (*boxed sectors*). Groups of unorganized vessels, developed from the outer tissues (mainly callus). **(l,n)** GFP visualized auxin response in auxin channels. Emergence of auxin channels was observed 3 DAW. Auxin response in “channel” cells was higher than that in surrounding neighbours. Day 4 and following days after wounding auxin channels developed along the first regenerated vessel strands. Broken arrows indicate wound.

New vessels developed also in deeply incised stems, in places immediately above the wound (Fig. 4g,h). In such areas, the local DR5 auxin response was always very high (Fig. 5g, inset). Regenerated vasculature was organized in groups of vessel-like cells, surrounded by DR5-positive neighbors (Fig. 4g,h, magnification). Connected vascular strands were never observed in this situation, the cells were often very short and shapeless (Fig. 4g,h), but still had some features characteristic for vessels, such as secondary cell walls and open perforations between neighboring cells (Fig. 4h). Small cell groups (2 or 3 cells) induced for circular vessel development could be recognized (Fig. 4i,j). In such cells, the *DR5::GUS* reporter activity was elevated and intrusive end growth was commonly recognized as very characteristic (Fig. 4i). Because of the typical morphological features, shape and length of these DR5-positive cells, and position within the tissues, these cells could be inferred to represent an early stage of the circular vessel development (Fig. 4i). In days 3 and 4 after wounding, first mature circular vessels were recognized (Fig. 4j) that were organized in closed rings and openings localized on lateral cell walls of individual cells connecting them with each other (Fig. 4j).

### Emergence of PIN1-mediated auxin channels and PIN1 polarity rearrangement

Analysis of vascular tissue regeneration implied changes in tissue polarity and creation of channels with elevated auxin response. To support these observations, we analyzed the cellular and tissue localizations of the PIN auxin transport proteins by means of anti-PIN1 antibodies. We observed significant differences in the cellular localization of PIN1 in nonwounded controls when compared to wounded inflorescence stems. In the controls, PIN1 was localized polarly in the vascular cambium, strictly at the basal plasma membranes of the cambial cells (Fig. 5a), whereas in the wounded stems, the PIN1 position changed gradually in tissues above and around the wound (Fig. 5b–d). At early regeneration stage (day 1 after wounding), PIN1 occurred primarily in an enlarged expression zone above the wound basically, in all cells predicted to form the auxin channels, but with an undefined polarity at this stage (Fig. 5b). Later on, the polar localization of PIN1 was gradually established and became restricted to the narrow strands corresponding to the new *PIN1*-expressing auxin transport channels in the regenerated areas (Fig. 5c,d). Thus, starting from day 2 and next after incision, PIN1 transiently moved from basal to lateral plasma membranes of the cells in emerged auxin channels that were either parallel to the cut (Fig. 5c) or around the wound, circumventing the cuts (Fig. 5d). Interestingly, in the close vicinity to the incision, the cellular position of PIN1 was often still undefined, namely the PIN proteins were localized both on lateral and basal plasma cell membranes (Fig. 5d). Figure 5e,f visualize tissue organization in Fig. 5c,d, respectively.

Changes in auxin response and auxin transport were also analyzed *in vivo*, in wounded stems of the Arabidopsis transgenic lines *pPIN1::PIN1:GFP*^15^ and *DR5::GFP*^9^. Tissue polarity changed rapidly around a wound (Fig. 5g–j). As monitored by the PIN1-GFP, new positions of PIN1 were gradually established in first 2 DAW. The originally undefined PIN1 localization at the plasma membrane of the wound-surrounding cells (Fig. 5g,h) gradually shifted to the lateral plasma membranes, starting from day 2 after wounding (Fig. 5i,j). DR5-GFP analysis revealed dynamic changes in auxin response around the incised regions of Arabidopsis stems (Fig. 5k–n). New DR5-positive auxin channels emerged 3 DAW and developed in regions above and around the wound (Fig. 5k,l). Threads of GFP-positive cells were also observed in outer tissues above the wound, in the vicinity of the forming callus (Fig. 5k,l). Moreover, groups of unorganized vessels regenerated from outer tissues and callus were well visible and developed above the wound (Fig. 5k). Completely regenerated vessel strands were found 4 DAW (Fig. 5m) and later on, positive auxin-responsive channels emerged along and parallel just regenerated vessel strands (Fig. 5n). Thus, reconstructed vasculature extended in the next few days after incision.

### Characteristics of the circular vessel formation after wounding

**Figure 6:**
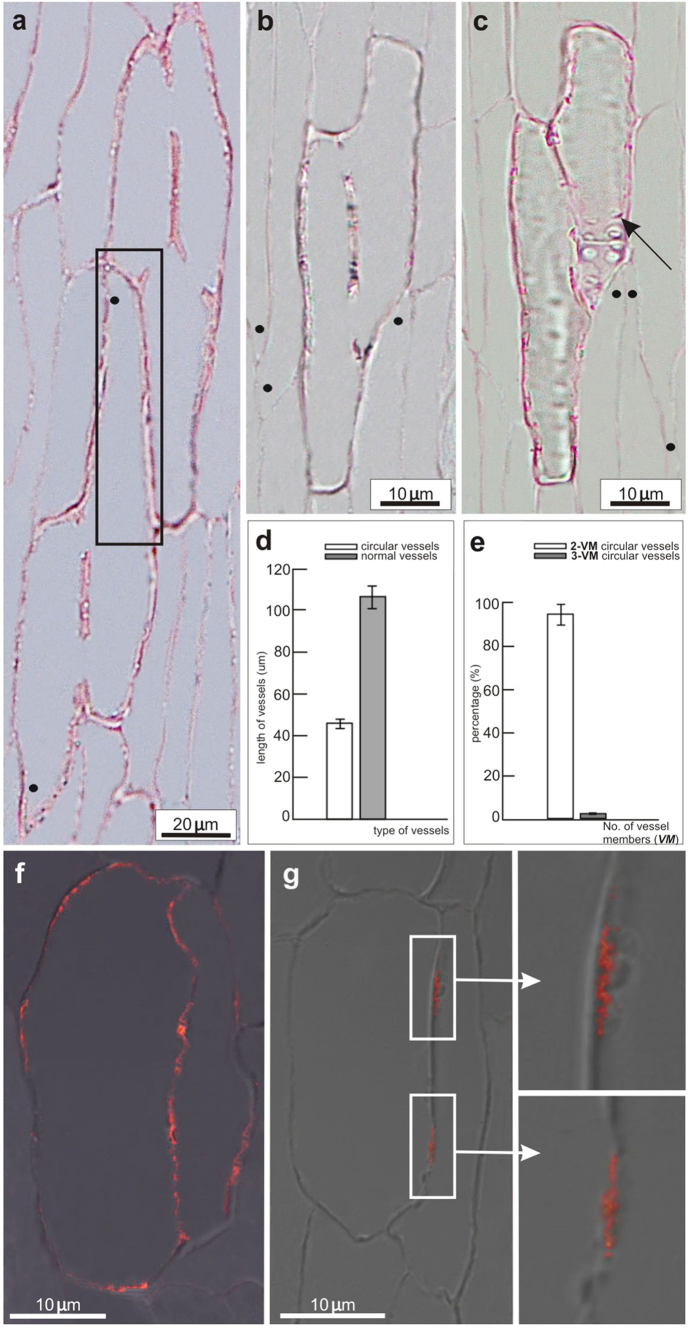
Development of circular vessels in wounded stems. **(a–c)** Wounded stems semi-thin tangential sections, 6 DAW. **(a)** Mature circular vessels consisting of two or three cells and arranged in a closed ring. Neighboring circular vessels developed independently and remained always separated, unconnected to each other (*boxed sector*). Intrusive growth of fusiform cambial cells differentiating into circular vessels was often reflected in mature forms of such vessels. Dots indicate intrusively grown ends. **(b)** Individual vessel cells are connected with each other by open perforations/enlarged pits localized on their lateral cell walls and surrounded by intrusively growing cells. **(c)** Secondary cell wall, the most characteristic feature for vessels allowing classifying them as a presumptive vessel type, is indicated by arrow. Dots mark intrusively growing cells. **(d)** Significantly different average length of circular vessels in wounded stems and normal vessels found in unwounded controls. The longitudinal vessels in controls were almost two-fold longer than the circular vessels in the interrupted stems. Significance was evaluated with unpaired Student *t*-test, *P* < 0.05. **(e)** Almost all circular vessels developing in incised Arabidopsis stems consisted of two vessel members (over 95% of all analyzed circular vessels). Three-cell circular vessels were very rare. Significance was evaluated with unpaired Student *t*-test, *P* < 0.05. **(f)** Wounded stem, 2 DAW. Localization of PIN1 auxin transporters was destabilized and gradually changed in circular vessels. At early stages of circular vessel development, the PIN1 position was nonpolar. Transporters moved from basal to the lateral and apical plasma membranes of two neighboring cells dedifferentiating into circular vessels. **(g)** Wounded stem, 4 DAW, later establishment of the new PIN1 polarity. Magnifications of boxed sectors showing that PIN1 signal was restricted to narrow, determined places on the lateral plasma membranes belonging to each of the neighboring cells.

Circular vessels occurred, in the regions above and in the immediate vicinity of the transversal cut. The emergence of the circular vessels in wounded Arabidopsis stems enabled us to assess their characterization by features typical for vessels and the polarity of individual cells that differentiated into circular vessels. Our observations showed that mature circular vessels differentiated from vascular cambium and that they consisted of 2 or 3 single cells connected with each other by lateral openings mostly localized on the lateral cell walls (Fig. 6a–c). However, neighboring groups of circular vessels did not connect with each other (Fig. 6a). Secondary cell walls were also detected in mature circular vessels (Fig. 6c). Interestingly, in analyzed Arabidopsis stems, growth was commonly intrusive in differentiating circular vessels and surrounding neighbors (Fig. 6a–c).

Single vessel cells that formed circular vessels seemed much shorter than those found in controls. Therefore, the average length of the circular vessels in wounded stems and normal (longitudinal) vessels in unwounded controls was also analyzed and compared (Fig. 6d). The length of vessels differed significantly: longitudinal, control vessels were more than twice as long as the circular vessels (109 μm and 47 μm average cell length, respectively). Over 90% of the circular or ring-formed vessels found in Arabidopsis were arranged as two and few as three individual cells dedifferentiated into vessel elements (Fig. 6e).

In the circular vessels, the PIN1 localization rearranged in an unprecedented way (Fig. 6f,g). At early stages of the circular vessel development (days 1 and 2 after wounding), PIN1 localized apolarly in differentiating cells, i.e. on their apical and lateral plasma membranes (Fig. 6f). At later stages (days 3 and 4 after wounding), the polar PIN1 localization was established (Fig. 6g) and PIN1 became restricted to the lateral plasma membranes. Intriguingly, the PIN1 signal was always observed in two distinct places at the plasma membranes, separated from each other and, moreover, each belonging to the other neighboring cell that differentiated into the circular vessel (Fig. 6g, magnifications).

## Discussion

De novo formation and regeneration of vascular tissues are instances of intercellular communication and coordinated tissue repolarization during developmental processes. More than 30 years ago, the canalization hypothesis had been proposed postulating the crucial role of the plant hormone auxin in these processes^5^,^6^. According to this hypothesis, position and patterning of new vasculature are established by the positive feedback between the auxin capacity and its flow directionality, leading to the establishment of canalized auxin channels. The exact molecular and cellular mechanisms underlying this feedback and emergence of polarized transport channels from the homogenous tissues are far from clarified. Regeneration of vasculature in response to wounding can best be studied in woody plants with active cylinders of vascular cambium and well developed secondary tissues^32^. However, studies in trees are limited because of the many experimental difficulties^31^. Thus, analysis of vascular tissue regeneration has been extended to the nonwoody dicotyledonous plants with primary tissue architecture, such as bean (*Phaseolus vulgaris*) or pea (*Pisum sativum*)^2^,^3^,^5^,^6^.

The major herbaceous model plant Arabidopsis is a well established model system for many molecular and genetic studies, including vascular development and secondary xylem formation^31^,^53^,^54^,^55^, because it can undergo secondary growth in hypocotyls^42^,^43^ and in inflorescence stems^44^,^45^,^46^,^47^,^48^. However, lack of some typical characteristics, such as cambial phenotypes, lack of secondary rays and variety of tracheary elements did not allow to employ Arabidopsis as a small “tree” with all vasculature features. Therefore, Arabidopsis could not be used to study some developmental processes that are characteristic to wood formation in trees and could not be regarded as a suitable model for research on the mechanisms of vascular cambium regeneration, vasculature reconstruction, and development of new vessels predicted by canalization/polarization concepts. Although the regeneration process in Arabidopsis has been analyzed tentatively^22^,^56^, it had never been studied in Arabidopsis stems with an active cylinder of vascular cambium and secondary tissue architecture analogous to the situation in trees. The use of Arabidopsis as a suitable model for vascular tissue and vascular cambium regenerations is, to our knowledge, possible only with a method introduced and described previously^39^. As described, an artificial weight (2.5 g) applied to the apical parts of immature inflorescence stems of Arabidopsis promotes secondary growth in the basal stem parts^39^. Thus, with this protocol we obtained stems with closed vascular cambium rings and all main features mimicking secondary growth in trees^40^,^49^. Furthermore, we show that analysis of vascular cambium regeneration, strictly correlated with new vasculature reconstruction, is also possible in wounded Arabidopsis stems, recommending Arabidopsis as a good model system to study coordinated tissue polarization during auxin canalization and tissue regeneration.

Auxin is regarded as a primary signal that induces vascularization *via* promotion auxin channel formation. The canalization hypothesis-predicted behavior is observed during de novo formation and regeneration of vasculature, especially of new regenerated vessels after incision, i.e. wounding or grafting^2^,^3^,^5^,^6^,^35^,^56^. However, the feedback mechanism between auxin and its flow and the vessel formation as a response to concentration gradients or directional auxin fluxes remains unclear^57^. Our results from Arabidopsis stems suggest successive changes in auxin response in wounded areas and sequences of cellular events accompanying the regeneration process in cambium.The locally elevated auxin response occurred already a few hours after wounding and gradually increased in these places. After a few days, a high auxin response appeared to mark the alternative way of the auxin flow to circumvent the wound. A presumptive new path of auxin flow was well visible in the auxin channels, namely cells with elevated auxin responses and subsequently differentiating into vessel elements. This observation fits very well with predictions from the canalization hypothesis, i.e. that auxin channels during regeneration canalize the auxin flow and have very high auxin levels^5^,^6^.

Originally, the feedback between auxin and its flow had been proposed to be realized by an auxin effect on the polar, subcellular localization of the PIN auxin transport proteins that, in turn, determine the auxin flow directionality^2^,^13^,^14^. Thus, auxin channel emergence can be visualized via rearrangements of the cellular position of PIN proteins. Indeed, in auxin-treated Arabidopsis roots, in wounded pea stems, or locally auxin-supplemented pea stems, the PIN polarity was rearranged in the two latter cases, marking the position of future vessel strands^2^,^3^. In wounded Arabidopsis stems with fully developed ring of vascular cambium, we also observed gradual changes in the PIN1 position, reflecting repolarization and de novo establishment of this tissue polarity in wounded areas. Initially, *PIN1* was expressed in cells around the wound whereafter auxin channels emerged and was localized less polarly at the plasma membranes. *PIN1* expression became increasingly stronger and more focus, when more defined auxin channels began to emerge. In a few days, the subcellular PIN1 position was gradually stabilized and restricted only to cell sides along the presumable direction of the auxin flow. Probably, this polarized PIN1 position marked the prospective perforation plates in fully regenerated vessels.

The impact of auxin on the polar PIN1 positioning might be part of a mechanism for auxin-regulated canalization^19^, possibly requiring local auxin accumulation that would trigger the TIR1-mediated Aux/IAA-ARF signaling pathway^58^,^59^ for both *PIN* expression and PIN polarity^2^,^60^. Another aspect of the auxin effect on the PIN polarity may be connected to the action of the Auxin-Binding Protein1 (ABP1) or related proteins. ABP1 is classified as an auxin receptor of which its action has at least in part been proposed to reside in the apoplast^20^ and that is required for polarization of puzzle-shaped epidermis cells^61^ and has an auxin effect on clathrin-mediated PIN internalization^17^,^18^ and on PIN polarity in root meristems^20^. However, recently, the originally reported embryo lethality of the *abp1* mutants has been shown to be due to mutations in the neighboring gene, necessitating additional analyses of the loss-of-function *abp1* phenotypes^62^. Clarifying contributions of the different auxin perception mechanisms and other molecular canalization components are now within reach by means of the extensive collection of mutant, transgenic, and marker lines in Arabidopsis.

In the course of our research on regeneration and canalization, we observed the interesting phenomenon of the so-called circular vessels that form isolated islands of differentiated vessel-like cells, indicative of small vortexes of circular auxin flow. Development of the circular vessels has been described previously^36^,^37^,^38^,^63^,^64^. Some features of the circular vessels, such as secondary cell walls and perforations^63^, resemble those of normal vessels. The occurrence of two perforation plates at the lateral side of the circular vessels supports their development through the auxin flow^63^, but the lateral openings could also be enlarged bordered pits developed between neighboring cells during their differentiation into circular vessels. Bordered pit fields have previously been referred to tracheary elements in trees^65^,^66^ and their development between adjacent cells is probably connected with local changes in cell wall composition, such as simultaneous dissolution and deposition of wall material^65^. The position of bordered pits during tracheid differentiation can be regulated by auxin and by its local accumulation in places of pit development^66^. Circular vessel development can be interpreted as the result of a circular auxin flow that establishes a circular polarity in (de)differentiating cells^36^. This intriguing scenario is supported by the asymmetric PIN1 localization in the differentiating circular vessels in Arabidopsis. PIN1 can be found at atypical positions in distinct, isolated patches at the plasma membranes of neighboring cells and such localization is in accordance with the suggested circular auxin flow and circular polarity of these cells. Thus, although these so-called circular vessels are often regarded as the out-of-function tracheary elements^38^, they might be spectacular examples of canalization that can be used to better understand (re)polarization processes. It remains entirely unclear by which mechanisms just a few (2 or maximum 3) cells are selected into the circular vessels for (de)differentiation, but this process involves obviously elevated auxin responses and atypical positions of the PIN1 auxin transporters.

**Figure 7:**
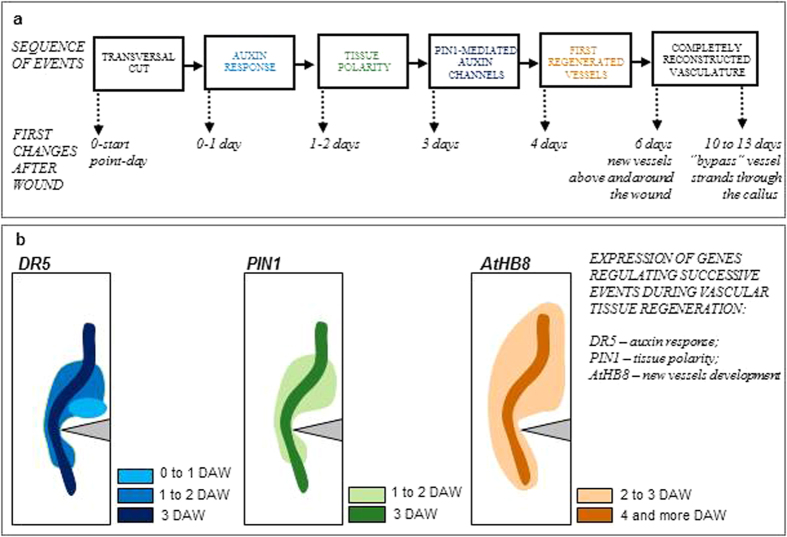
Summarizing model for the successive cellular events during the vascular tissue regeneration in wounded Arabidopsis stems. **(a)** Sequence of events in the transversal cut region. Elevated auxin responses and tissue repolarization were visible within 24 h after wounding, whereas new established tissue polarity and PIN1-mediated auxin channels emerged in next two days. Four days after wounding (DAW), the first vessel strands developed above and around the cut. Completely regenerated vasculature was observed starting from the day 6. Later on, 10 to 13 DAW regenerated “bypass” vessel strands developed through callus. **(b)** Expression of genes monitoring the auxin response (*DR5*), the tissue polarity (*PIN1*), and new vessel development (*AtHB8*) varied during vasculature regeneration. *DR5* expression and cellular auxin response slightly preceded *PIN1* expression, new tissue polarity establishment, and PIN1 transporter relocalization. The earliest regenerated vessels developed 4 DAW. The developmental places were indicated by the *AtHB8* marker gene expression.

Here we show that Arabidopsis comes out as a suitable model system for the analysis of vascular cambium regeneration that typically occurs in trees and provides the possibility to study this spectacular example of coordinated tissue differentiation and repolarization (Fig. 7). This process is accompanied with temporal and spatial events following new vessels development: (i) increase in local auxin responses in tissues above and around the wound; (ii) emergence of auxin channels marked by elevated auxin responses; (iii) tissue repolarization and de novo establishment of new polarity through the changed position of the PIN1 auxin transporters at plasma membranes of differentiating cells; (iv) development of new vessel strands along and around the wound; and (v) emergence of the phenomenon of the circular vessels in the vicinity of the wound. These processes were visualized by means of the auxin response marker *DR5*, the auxin transporter *PIN1*, and the vessel identity marker *AtHB8*.

## Methods

### Plant material and growth conditions

Wild-type *Arabidopsis thaliana* (L.) Heynh. ecotype Columbia 0 (Col-0) and transgenic lines *DR5::GUS*^52^, *DR5::GFP*^10^, *pPIN1::PIN1:GFP*^15^, and *AtHB8::GUS* in Wassilevskija (Ws) background^50^ were used. Seeds were obtained from Nottingham Arabidopsis Stock Centre (NASC, http://www.arabidopsis.info/BasicForm). Individual plants were grown in pots with a mixture of soil and vermiculite (1:1, v/v) in growth chambers and under stable conditions as described^40^. Each marker and conditions were analyzed at least twice on total of 432 inflorescence stems of Arabidopsis.

### Design of the experiments

The experiments were divided in two critical steps. *I STEP* was done to obtain a closed ring of active vascular cambium and secondary tissue architecture in immature inflorescence stems (Supplementary Fig. S4a,b). This part of the experiment had been described previously^40^. Briefly, plants with immature inflorescence stems (9 to 10 cm tall) were used. Such stems were characterized by the primary tissue architecture, i.e. vascular bundles separated by interterfascicular parenchyma sectors. Stems were decapitated with a sharp razor blade, the apical floral parts (1 to 2 cm) were removed, and the artificial weight, a 2.5-g lead ball connected with a plastic tube was applied (Supplementary Fig. S4a,b). Decapitated stems covered by the artificial weight were additionally supported by a wood stick to avoid their bending. With this method, secondary tissue architecture could be obtained in a short time (merely 6 days after weight application) in the basal parts of previously immature Arabidopsis stems (5-mm segments above the rosette). Active vascular cambium cylinder and secondary tissues, namely phloem and xylem, mimicked vascular tissues in woody plants (Supplementary Fig. S4b, shadow frame). In total, 432 plants were analyzed at this step.

*II STEP* was done to analyze regeneration of incised vascular cambium and formation of new vessels in wounded stems (Supplementary Fig. S4c). Inflorescence stems were cut precisely with a sharp razor blade 3 to 4 mm from the rosette in the transversal plane of the basal sectors with vascular cambium and secondary tissues to interrupt their longitudinal continuum (Supplementary Fig. S4c). Plants were still covered with the artificial weight during this experimental step. Axillary buds grown above the rosette leaves were not removed, thus remaining the source of endogenous auxin (Supplemental Figs S1 and S4a,c). Vasculature regeneration was analyzed in stems with fully developed, closed cambial rings, and secondary tissues in their basal parts. Therefore, at this stage, stems were evaluated and selected for further analysis as follows: (i) after this step, basal stem parts were sectioned manually (2 to 3 hand-cut transverse sections were made); (ii) stained with 0.1% (w/v) Safranin O solution for a quick lignin test in the cell walls; and (iii) analyzed with a stereomicroscope to select stems with closed vascular cambium rings and secondary growth. Only stems selected in this manner were used to study vasculature regeneration. The efficiency of the method was very high. In over 88% of the weight-applied stems, the closed rings of secondary tissues were found and, among them, almost all stems were selected with fully regenerated vasculature after wounding (Supplementary Fig. S4c, shadow frame). In this step, 383 plants were analyzed.

In both experimental steps, immature stems without weight application were used as controls for the mechanical stimulus and weight-applied and nonwounded stems as controls for transversal cuts. Moreover, uncut mature inflorescence stems (>25 cm tall) were analyzed as well and used as additional controls for stems with weight-induced secondary growth. In mature stems, secondary growth developed in the basal parts. These observations and statistical analyses were done on a total of 46 mature plants (Supplementary Fig. S2).

### Histological procedures

For detailed histological examination, 3-mm basal segments of the wounded stems were processed by fixation, embedding, and sectioning as described^40^. Briefly, first, the samples were fixed with 2% (w/v) glutaraldehyde (Sigma-Aldrich) in phosphate buffer (pH 7.4) at 4 °C, overnight; then, they were washed in phosphate buffer for 3 × 15 min and dehydrated with successive water solutions of 99.9% (w/v) ethanol (graduate percentages 5% to 100%), 30 min for each change at room temperature (RT); afterward, they were infiltrated with propylene oxide (Serva) for 2 × 1 h at RT and with solutions of propylene oxide/PolyBed 812 epoxy resin (3:1, 1:1, 1:3, v/v) at 4 °C overnight; and finally, they were transferred to silicon-embedding molds and polymerized with 100% PolyBed 812 resin (Polysciences) at 35 °C, 45 °C, 60 °C, each temperature for 24 h. Polymerized samples were cut with an ultramicrotome (Leica EM UC6) into semi-thin sections (2-μm thick) with the glass knife, attached to microscope slides covered by Haupt’s adhesive and stained with Periodic Acid-Schiff’s + Toluidine Blue O^67^. Lastly, the series of semi-thin sections were examined, section by section to visualize changes in the wounded areas and the regeneration process. On average, series of 30 to 40 semi-thin (2-μm thick) sections were analyzed and compared, allowing a precise analysis of vessel connections in regenerated vasculature (presented as one representative picture in the figures).

### Histochemical assays for GUS activity

The histochemical analysis was done as described previously^52^,^68^. Samples were incubated with X-Gluc solution at 37 °C, overnight, and fixed with a 99.9% ethanol/99.6% acetic acid (9:1, v/v) solution at 4 °C overnight. The samples with positive GUS reaction were embedded in LR White acrylic resin (Polysciences) and then infiltrated with series of LR White/99.9% ethanol solutions (3:1, 1:1, 1:3, v/v) at 4 °C overnight, polymerized in gelatin capsules with 100% LR White at 50 °C for 24 h. Washing and dehydrating steps were omitted in this procedure.

### Immunolocalization of PIN1 protein

Immunolocalization was done as described previously^69^. The PIN1 position was monitored with the anti-PIN1 antibody visualized by a fluorescence-labeled Cy3 secondary antibody^17^. For this procedure, samples were embedded in LR White acrylic resin^40^, subsequently cut with the ultramicrotome (Leica EM UC6) and the diamond knife in a series of semi-thin sections (1-μm thick), attached to microscope slides covered by Poly-l-lysine (Menzel-Glaser), and processed for immunolocalization. Briefly, slides were washed with phosphate buffered saline (PBS) (pH. 7.4), 2 × 15 min at RT, incubated first with 2% (w/v) blocking solution (bovine serum albumin in PBS) for 30 min at RT and then with a primary antibody (purified rabbit anti-PIN1 antibody, dilution 1:1000) in the humid chamber at 4 °C overnight. Then, the slides were washed with PBS, 5 × 10 min at RT, incubated with the fluorescence-labeled secondary antibody (goat, fluorescence-labeled anti-rabbit-Cy3, dilution 1:600) for 4 h at RT, again washed in PBS, 5 × for 10 min at RT. Finally, they were mounted with antifade FluoroMount Aqueous Mounting medium (Sigma-Aldrich) and analyzed with an epi-fluorescent or confocal laser-scanning microscope.

### Analysis of GFP signal *in vivo* in cells

Changes in expression of genes regulating auxin response and tissue polarity were analyzed *in vivo*. The *DR5::GFP* and *pPIN1::PIN1:GFP* transgenic lines were used. Sections from non-fixed samples of incised stems were handly cut with a sharp razor blade, mounted in 25% (w/v) glycerol solution, and analyzed with a confocal laser-scanning microscope.

### Statistical analyses

Statistical significances were evaluated with the Student’s *t*-test (unpaired Student *t*-test, *P* < 0.05). Transition from primary to secondary tissue architecture in weight-applied immature stems was analyzed on a total of 432 plants. Differences in vessel lengths were compared: 100 individual cells from both vessel types (normal vessels in unwounded controls and regenerated vessels/circular vessels, in wounded stems) were selected and measured along the tangential dimensions. Additionally, regenerated vessels of 383 wounded stems were analyzed. The number of vessel cells in circular vessels was evaluated. In total, 156 plants with circular vessels were found in wounded stems. Statistical analyses were done with Office Excel 2003 (Microsoft).

### Microscopy

Samples of wounded stems were analyzed with a stereomicroscope (NIKON MSZ1500) equipped with the charge-coupled device camera (DS-Fi1). Semi-thin sections were examined with a transmitted light microscope OLYMPUS BX41 equipped with fluorescence filters. PIN1 localization visualized by fluorescence-labeled anti-rabbit Cy3, and auxin response in wounded areas was analyzed using a confocal laser-scanning microscope ZEISS LSM5 Exciter or Zeiss Observer.Z1. Fluorescence of Cy3 and GFP (reporter lines *DR5::GFP* and *pPIN1::PIN1:GFP)* was excited with a multiband argon laser at wavelength of 561 nm and 488 nm, respectively. The acquired images were processed with ZEN 2008 and ZEN 2012 Light Edition softwares.

## Additional Information

**How to cite this article**: Mazur, E. *et al.* Vascular cambium regeneration and vessel formation in wounded inflorescence stems of Arabidopsis. *Sci. Rep.* **6**, 33754; doi: 10.1038/srep33754 (2016).

## Supplementary Material

Supplementary Information
