# Supplementary material for: Vascular cambium regeneration and vessel formation in wounded inflorescence stems of Arabidopsis
Source: Sci Rep. 2016 Sep 21;6:33754. doi: 10.1038/srep33754 (PMC5030676; doi:10.1038/srep33754)
Supplement: Supplementary Information [file srep33754-s1.pdf]

SUPPLEMENTARY INFORMATION

Vascular cambium regeneration and vessel formation in wounded inflorescence stems of Arabidopsis

Ewa Mazur, Eva Benková & Jiří Friml

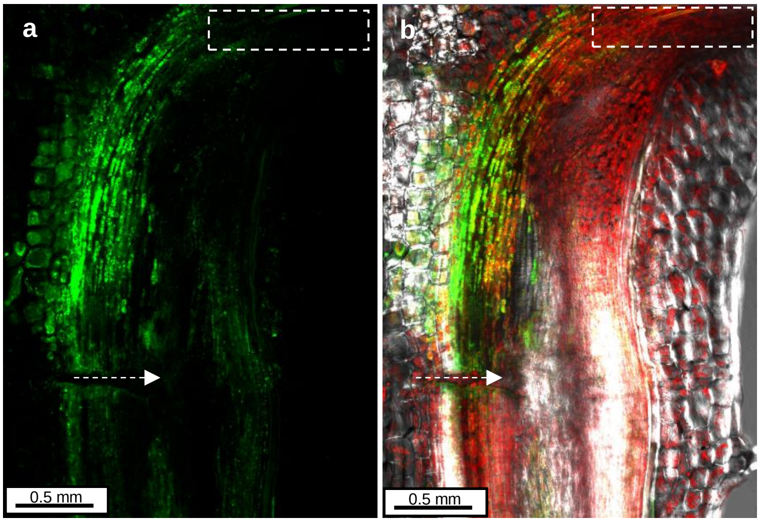

Supplementary Figure S1. Axillary buds as a source of endogenous auxin.

(a) *DR5::GFP* transgenic line, confocal image of hand-cut tangential sections of wounded stem. Axillary buds that developed above the rosette were not removed and served as endogenous auxin source (*boxed sectors*). The polar auxin flow between axillary buds and the wound was not disrupted, auxin flow from the axillary bud through tissues above the wound in polar direction and was finally around the wound (as visualized by *DR5::GFP*). (**b**) Transmitted light image for tissue arrangement in **a**. The broken arrows indicate wound.

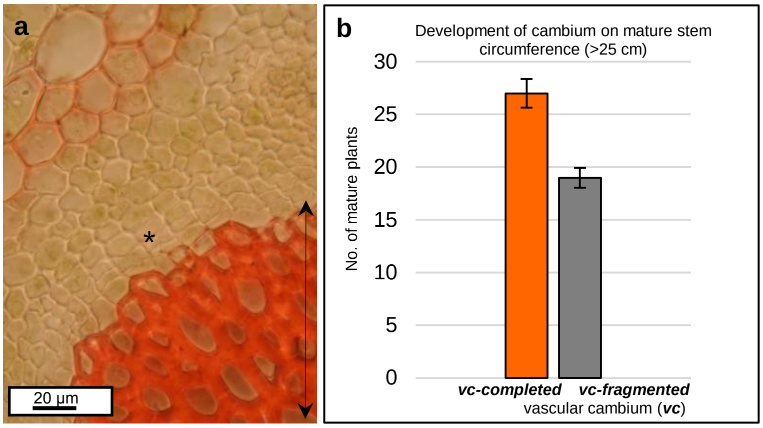

Supplementary Figure S2. Secondary growth in mature inflorescence stems (>25 cm tall).

(a) Wild type Col-0, Safranin O-stained hand-cut transverse section through the basal parts of mature stems with secondary vascular architecture. Such mature stems were obtained after more than 2 months after inflorescence stem emergence. The basal sectors with secondary vascular tissues were 2, to maximum 3, mm thick. Arrow indicates tracheary elements and enhanced amount of interfascicular fibers. Asterisk indicates cambium. **(b)** Closed cambial rings observed in over 50% of mature stems. Therefore, in more than 40% of the stems, secondary growth was fragmented around the stem circumference (*n* = 46 mature plants; error bars, SD of the mean).

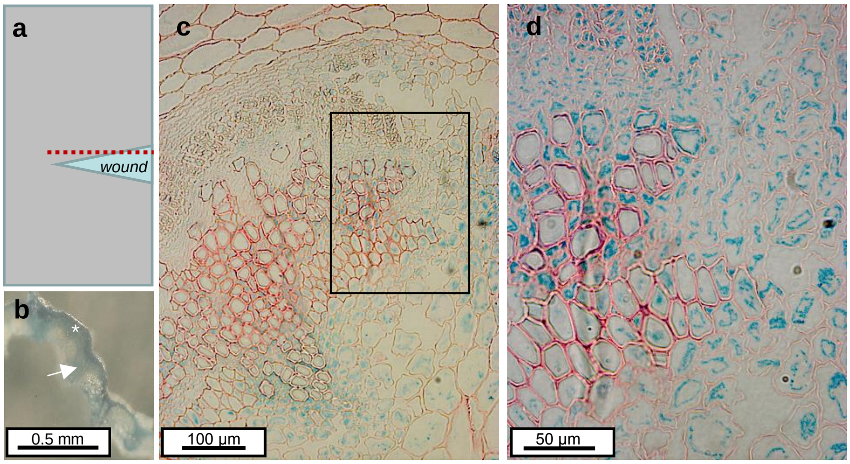

Supplementary Figure S3. Transverse sections through wounded stem regions.

(a) Schematic view of the stem wounded in a transversal plane reflecting the situation in **c, d**. Broken line indicates the plane of cutting. **(b)** *DR5::GUS*, hand-cut transverse section through the wounded region, 3 DAW. The DR5::GUS reaction was visible in vascular bundles and outer tissues, extending to the cortex and epidermis. Asterisk indicates outer tissues. Arrowhead indicates vascular bundle. **(c,d)** *DR5::GUS*, semi-thin transverse sections through wounded areas, 4 DAW. Sections were made on the border wound/regenerated vasculature, as schematically shown in **a**. **(c)** The auxin response was very high in almost all tissues of the wounded region. **(d)** Magnification of the boxed sector in **c**, in the nearest wound neighborhood. Tissues were poorly recognizable, because wounded tissues were mixed with callus cells in the sectioning plane.

Critical steps	I STEP		II STEP
	**a**/decapitation of apical parts of stem	**b**/application of artificial weight (2.5g)	**c**/transversal cut
Design of experiments	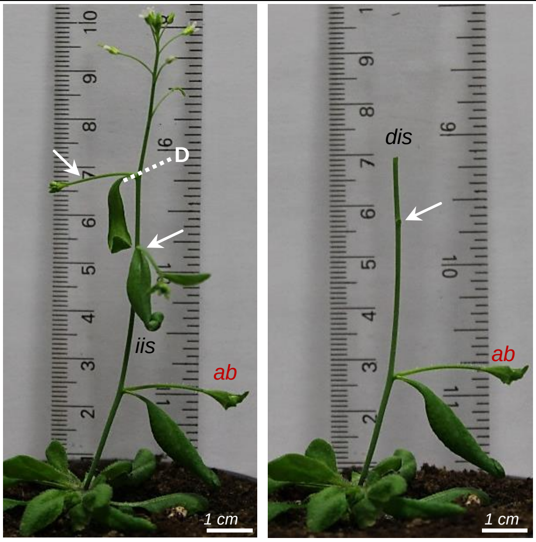<br>**D** – place of decapitation;<br>*iis* – immature inflorescence stem (9-10 cm tall);<br>*dis* – decapitated inflorescence stem (7-8 cm tall);<br>***ab*** – axillary bud as a source of exogenous auxin<br>upper axillary budsemoved for weight application are indicated by *arrows*	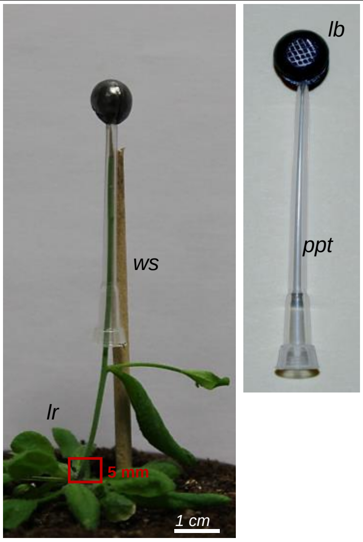<br>*lr*- leaf rosette;<br>*lb* – leaden ball (2.5 g);<br>*ppt* – polypropylene tube (=pipette tips);<br>boxed sector – 5-mm sector of stems above *lr* with the secondary growth	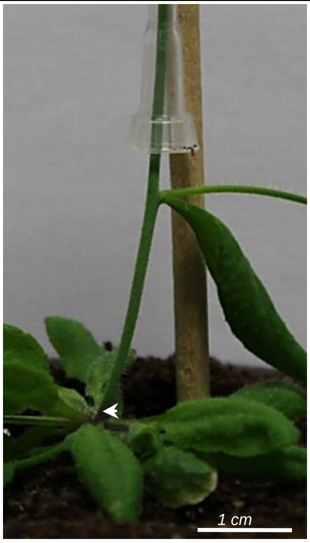<br>place of transversal cut (3-4 mm from leaf rosette) is indicated by *arrowhead*
Changes in analyzed parts of inflorescence stems	Primary tissue architecture in basal part of the stems:<br>-fascicular sectors with collateral vascular bundles (*vb*);<br>-interfascicular sectors with parenchyma cells between *vb*	Secondary tissue architecture in basal parts of the stems (5-mm sectors above *lr*) **mimicking vasculature in woody plants**:<br>-close ring of vascular cambium on stem circumference;<br>-variety of cambial phenotype with two types of cambial cells: fusiform cambial cells and ray cambial cells;<br>-intrusive growth of fusiform cambial cells;<br>-secondary tissues differentiated from cambial derivatives;<br>-variety of tracheary elements	Regenerated vascular tissue in the neighborhood of transversal cut:<br>-new vessel strands above and around the wound;<br>-"bypass" strands through callus;<br>-circular vessels in the neighborhood of the wound
Waiting time	**2-3 weeks** after germination (9-10-cm *iis*)	**6 days** after artificial weight application	**6 days** after transversal cut
Number of analyzed plants	**432 plants**	**432 plants**	**383 plants**
Frequency of changes in analyzed plants	-	**88,7% of plants** with the secondary growth (vascular cambium and secondary tissues on the stem circumference)	**99,2% of plants** with regenerated vascular tissue above or around the wound (including circular vessels and "bypass" strands)

Supplementary Figure S4. Experimental design to obtain secondary tissue architecture and vascular tissue regeneration in immature inflorescence stems of Arabidopsis.
(a,b) Step-I experiments to obtain a closed ring of active vascular cambium and secondary tissues in immature inflorescence stems. **(c)** Step-II experiments to obtain vascular cambium regeneration in incised stems and new vessels development. Details of the experimental design are included in the figure.
